# Microbial Biosurfactants: Antimicrobial Activity and Potential Biomedical and Therapeutic Exploits

**DOI:** 10.3390/ph17010138

**Published:** 2024-01-22

**Authors:** Patricia Puyol McKenna, Patrick J. Naughton, James S. G. Dooley, Nigel G. Ternan, Patrick Lemoine, Ibrahim M. Banat

**Affiliations:** 1 The Nutrition Innovation Centre for Food and Health (NICHE), School of Biomedical Sciences, Faculty of Life and Health Sciences, Ulster University, Coleraine BT52 1 SA, UK; puyol_mckenna-p@ulster.ac.uk (P.P.M.); pj.naughton@ulster.ac.uk (P.J.N.); jsg.dooley@ulster.ac.uk (J.S.G.D.); ng.ternan@ulster.ac.uk (N.G.T.); 2 Nanotechnology and Integrated Bioengineering Centre (NIBEC), School of Engineering, Ulster University, Belfast BT15 1ED, UK; p.lemoine@ulster.ac.uk; 3 Pharmaceutical Science Research Group, Biomedical Sciences Research Institute, Ulster University, Coleraine BT52 1SA, UK

**Keywords:** biosurfactants, antibiotic resistance, antibiofilm, COVID-19, anti-cancer

## Abstract

The rapid emergence of multidrug-resistant pathogens worldwide has raised concerns regarding the effectiveness of conventional antibiotics. This can be observed in ESKAPE pathogens, among others, whose multiple resistance mechanisms have led to a reduction in effective treatment options. Innovative strategies aimed at mitigating the incidence of antibiotic-resistant pathogens encompass the potential use of biosurfactants. These surface-active agents comprise a group of unique amphiphilic molecules of microbial origin that are capable of interacting with the lipidic components of microorganisms. Biosurfactant interactions with different surfaces can affect their hydrophobic properties and as a result, their ability to alter microorganisms’ adhesion abilities and consequent biofilm formation. Unlike synthetic surfactants, biosurfactants present low toxicity and high biodegradability and remain stable under temperature and pH extremes, making them potentially suitable for targeted use in medical and pharmaceutical applications. This review discusses the development of biosurfactants in biomedical and therapeutic uses as antimicrobial and antibiofilm agents, in addition to considering the potential synergistic effect of biosurfactants in combination with antibiotics. Furthermore, the anti-cancer and anti-viral potential of biosurfactants in relation to COVID-19 is also discussed.

## 1. Introduction

The misuse of antimicrobial agents, combined with the lack of recent drug development, has accelerated the appearance of antibiotic-resistant pathogens [1]. Over the past two decades, methicillin-resistant *Staphylococcus aureus* has become a major public health concern; simultaneously, the emergence of new resistant pathogens poses a serious threat to the healthcare environment [2]. Infections caused by *Enterococcus faecium*, *Staphylococcus aureus*, *Klebsiella pneumoniae*, *Acinetobacter baumannii*, *Pseudomonas aeruginosa*, and *Enterobacter* spp. (ESKAPE pathogens) provide significant challenges in terms of resistance, as they can exhibit multiple resistance mechanisms and possess the capacity to transfer resistance through horizontal gene transfer [3]. In an attempt to accelerate the fight against antimicrobial resistance, the Centers for Disease Control and Prevention (CDC) has developed an in-depth report highlighting the most prevalent resistant organisms, classifying them into three threat categories (urgent, serious, and concerning) based on the level of pathogenicity (Table 1) [4]. One of the strategies proposed to reduce the number of antibiotic-resistant infections is combining antimicrobials with other compounds, such as biosurfactants [5].

Biosurfactants are natural amphiphilic compounds, synthesized as secondary metabolites by bacteria, yeasts, and fungi, which possess surface activity [6]. These organisms can secrete biosurfactants as a byproduct during the biodegradation of complex substrates such as hydrocarbons, facilitating the solubilization and utilization of these substrates and thereby enhancing microbial degradation capabilities [7]. Subsequently, when biosurfactants are dissolved, they are capable of lowering the surface tension of the solution, resulting in them efficiently adsorbing to surfaces. These compounds present many advantages over chemical surfactants, including low toxicity, high biodegradability, environmental compatibility, and specific activity at extreme temperatures, pH, and salinity, and as a result, exhibit higher stability than synthetic surfactants [8,9]. Given these properties, biosurfactants have been studied as potential substitutes for some products in the pharmaceutical, healthcare, cosmetics, and detergent industries [10,11,12]. Their inherent stability has proven capabilities to enhance the chemical and physical properties of formulations, for example, improving solubility while reducing phase separation and aggregation and consequently extending the shelf life of biosurfactant-containing products [13]. In addition to their uses in the biomedical field, these compounds are also utilized in a number of industrial and environmental applications, such as bioremediation and oil recovery [14,15]. Nevertheless, large-scale production and mass application of biosurfactants are limited by relatively low yields and associated high production costs [16]. Ceresa et al. (2023) have emphasized that a significant challenge encountered by the biomedical and pharmaceutical industries in utilizing biosurfactants is ensuring compound purity and/or types and proportions of the different congeners produced. This is due to the fact that biosurfactants are typically generated as a combination of different congeners, or slightly different chemical variations in terms of acetylation or bonds, each having unique characteristics [17]. An example of this can be found in lipopeptides, a type of low-molecular-weight biosurfactant, which display a wide range of structures and functions. Despite the significant growth in lipopeptide research in recent years, approximately 30% of bacterial lipoproteins remain functionally uncharacterized [18]. Another limitation observed in the large-scale production of biosurfactants is the low yields possible for most. The absence of standardized methodologies, coupled with the limited yields of microbial fermentation, has slowed down their commercialization process. The optimization of biosurfactant production relies on crucial production parameters, including the pH of the medium, incubation temperature, oxygen availability, and nutrient composition [17]. Exploring efficient and affordable sources of carbon and nitrogen, optimizing the growth medium, and utilizing genetically modified strains are a few strategies that can enhance yields and subsequently decrease production expenses [19].

The specific antimicrobial mechanism(s) of biosurfactants remains unclear; however, it is suggested that biosurfactants interact with bacterial cell membranes but are not limited to a single mechanism [2,20,21]. It has been proposed that rhamnolipids bind to the bacterial membrane via electrostatic interactions [22] between the polar groups of the positively charged surfactant and the negative charges of some of the molecules that form bacterial membranes (for example, lipopolysaccharides in Gram-negative bacteria and lipoteichoic acid in Gram-positive bacteria) [23]. Another hypothesis is that the alkyl chains of the surfactants interact with the lipid bilayer of the membrane through hydrophobic interactions [24], disrupting the membrane architecture and allowing the transport of intracellular constituents across the membrane [25], resulting in cytoplasmic leakage and consequently in cell death [26].

## 2. Classification of Biosurfactants

**Figure 1:**
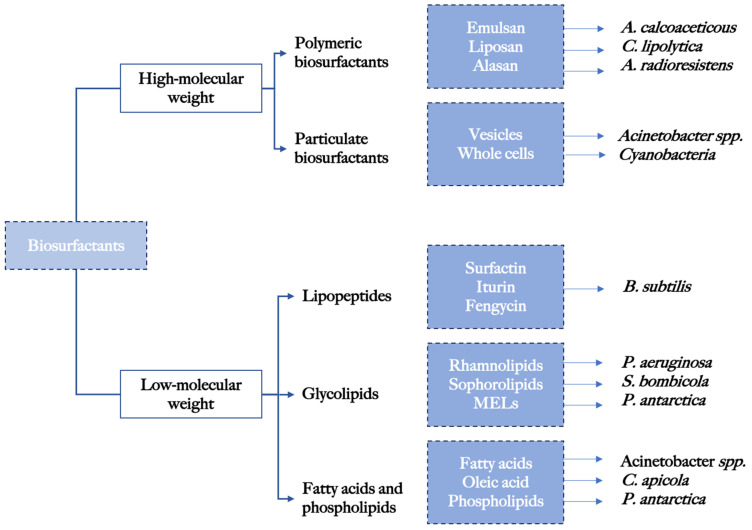
Classification of biosurfactants including most prevalent producing microorganism.

Surface-active compounds are classified into high-molecular-weight compounds (polymeric biosurfactants) and low-molecular-weight compounds (glycolipids and lipopeptides) depending on structure [27], producing organism, and molecular weight (Figure 1). Low-molecular-weight biosurfactants are efficient emulsifiers [28] whereas high-molecular-weight biosurfactants are capable of reducing surface and interfacial tensions [29] and have become a matter of specific interest in recent years in relation to their antimicrobial and antibiofilm properties [30].

Lipopeptide biosurfactants are cyclic or linear structures made up of hydrophilic peptide sequences that are usually seven to ten amino acids long, with a fatty acid chain as the hydrophobic component and mainly produced by species of the genus *Bacillus* [31].

Glycolipids are carbohydrates of the mono-, di-, tri-, and tetrasaccharide types including glucose, mannose, galactose, rhamnose, or glucuronic acid, linked to one or two fatty acid chains [32]. They are divided into different groups according to their structure. Examples include rhamnolipids, produced by some species of the genus *Pseudomonas* and *Burkholderia* [33], mannosylerythritol (MELs), a glycolipid produced by yeasts of the genera *Pseudozyma*, *Ustilago,* and *Candida* [8] and sophorolipids, synthesized by *Candida* species [34].

Rhamnolipids are low-molecular-weight secondary metabolites of glycolipids produced mainly by species of the genus *Pseudomonas* and *Burkholderia*, although *P. aeruginosa* is the predominant rhamnolipid-producing bacterial species. These compounds consist of a hydrophilic head, consisting of one or two rhamnose sugar molecules, linked by an o-glycosidic bond to a hydrophobic tail, consisting of one or two fatty acid chains [33]. *Pseudomonas* spp. synthesize two types of rhamnolipids, depending on the number of rhamnose residues in their structure, into mono-rhamnolipids (one rhamnose) and di-rhamnolipids (two rhamnose). Naturally occurring rhamnolipids are usually found in the form of mixtures of different congeners, i.e., both mono- and di-rhamnolipids, differing in fatty acid chain structures, varying between C8 and C16 [35]. Using mass spectrometry, more than 30 different congeners of rhamnolipids have been confirmed produced by *P. aeruginosa* to date [36]. During the last decades, rhamnolipids have been the subject of study due to their surfactant and physicochemical properties. They exhibit a significant surfactant activity, with the ability to reduce water surface tension from 72 mN/m to less than 30 mN/m [37].

Sophorolipids, the second most reported glycolipid biosurfactant, are produced by a few specific yeasts, such as *Starmerella bombicola* and *Candida apicola*. They can be lactonic or acidic and have varying levels of acetylation on the sophorose moiety. The hydroxy fatty acid component, which typically has 16 and 18 carbons, can vary in chain length, saturation level, and hydroxylation position (terminal or subterminal) [38]. Similar to rhamnolipids, sophorolipids also present high surfactant activity, reducing the surface tension of water from 72 mN/m to 40 mN/m, both acidic and lactonic [39].

## 3. Antimicrobial and Antibiofilm Activity of Biosurfactants

Biosurfactants comprise a group of unique amphiphilic molecules of microbial origin that are capable of interacting with lipidic components of microorganisms, altering their physicochemical properties [11]. It has been established that several of these compounds exhibit biological properties (antimicrobial and antifungal activity) [40] that make them potentially suitable for use in medical, pharmaceutical, and agricultural applications [41].

As previously stated, biosurfactants are distinguished by their low toxicity and high biodegradability, particularly as their chemical structures are simple sugars and fatty acids or polypeptide components. These properties ensure the safety of drug formulations and reduce the likelihood of adverse effects, while also preserving the effectiveness of bioactive substances. Surfactin, a lipopeptide synthesized by the microbial species *B. subtilis*, has been suggested as a potentially advantageous substitute in detergent and soap formulations. As reported by Fei et al. (2019), surfactin presents low-toxic non-irritant properties that make it versatile in many everyday household products. In addition to the primary irritation index (PII = 0), they demonstrated that when testing acute oral toxicity, surfactin presented low toxicity compared to synthetic surfactants (LD_50_ > 5000 mg kg^−1^) highlighting its potential use in drug formulations [42].

Many studies can be found in the literature demonstrating not only the antibacterial and antifungal properties of several biosurfactants but also their effect on biofilm disruption and inhibition. Glycolipids have been reported to show antibacterial activity against both Gram-negative and Gram-positive microorganisms including *S. aureus*, *E. coli*, *K. pneumoniae,* and *B. subtilis* by causing cytoplasmic membrane damage [43]. However, as demonstrated by de Freitas Ferreira et al. (2019), the antimicrobial activity of rhamnolipids against Gram-positive bacteria was favored under more acidic concentrations [44]. The antimicrobial activity of sophorolipids has also been demonstrated throughout the literature. Diaz de Rienzo et al. (2018) investigated both the antimicrobial properties and biofilm disruption of sophorolipids obtained from *Candida bombicola* ATCC 22214 in Gram-negative and Gram-positive bacteria. It was demonstrated that, at low concentrations (50 g L^−1^), sophorolipids exerted a fungicidal effect as well as biofilm disruption at the same concentration [45]. Despite these advantages and the extensive use of these surface-active agents, their applicability in the medical field is still very limited [46]. In this context, innovative approaches are being developed to improve the multifunctionality of biosurfactants in order to broaden the field of applications [47]. The development of new strategies based on biosurfactants offers the opportunity to further expand the areas of application [2]. Of particular interest is their use in combination with antibiotics, with the aim of achieving synergistic effects in the activity against various pathogenic microorganisms [48].

### 3.1. Synergistic Effect of Antimicrobials and Biosurfactants

As an example of the synergistic effect of biosurfactants and antibiotics, Shusterman et al. (2021) demonstrated that for *E. coli*, the rhamnolipids tested using disc diffusion assay increased the zone of inhibition of four out of six antibiotics tested (ampicillin, chloramphenicol, erythromycin, kanamycin, and tetracycline). Moreover, when tested against *Bacillus megaterium*, an increase in the zone of inhibition was reported for all six antibiotics tested [49]. Regarding sophorolipids, it is hypothesized that the synergistic effect with antibiotics is achieved due to the hydrophobic surface of the biosurfactant. Its interaction with the microorganism’s lipid bilayer increases permeability, allowing antimicrobial agents to enter the microbial cells easily and therefore increasing the antibiotic’s efficacy [16]. In the study carried out by Juma et al. (2020) in liquid culture, it was demonstrated that the combined use of tetracycline and sophorolipids at sub-critical micelle concentrations proved to reduce bacterial growth greatly compared to the treatment with tetracycline alone, causing morphological changes in the bacterial cell and inducing cell damage [5]. A further example of the joint effect of combining biosurfactant and antibiotic treatment can be observed in the investigation carried out by Amirinejad et al. (2023). GBB12, a glycolipid synthesized by *Shewanella algae*, was shown to possess notable antimicrobial properties both independently and when used in conjunction with ciprofloxacin and gentamicin. The findings demonstrated that GBB12 by itself effectively inhibited the formation of biofilms caused by *MRSA* and *A. baumanii*, with inhibition rates ranging from 84% to 93%. However, when used in conjunction with gentamicin or ciprofloxacin, an effectiveness of 99% was achieved in disrupting biofilms. It was noted that the glycolipid induced cell membrane disruption, leading to leakage of cytoplasm and subsequent cell death [50].

### 3.2. Biosurfactants in Biofilm Inhibition

Currently, one of the main causes of microbial infections and the development of resistance is the presence of biofilms [51]. When a microorganism is deposited on a certain surface, a structured biological community or bacterial biofilm is formed [52]. This biofilm is self-regulated by quorum-sensing molecules and grows enveloped in an extracellular matrix which protects it from the environment, prevents the action of antimicrobial agents, and, consequently, greatly hinders their elimination [53]. Recent studies have shown that approximately 80% of chronic and recurrent microbial infections are caused by biofilms [54]. There are numerous studies in the literature showing the promising effects of biosurfactants on biofilms, demonstrating that they can reduce and inhibit the formation of these. As an example, Turbhekar et al. (2015) demonstrated that the adhesion of biofilms to microtiter plates was reduced by up to 50% when treating plates with a rhamnolipid solution at 5%. They also observed that at similar concentrations, the rhamnolipid solution was able to remove biofilms that had already formed [55]. Furthermore, Rivardo and coworkers (2009) reported conclusive results when measuring the anti-adhesive activity of the biosurfactant obtained from *B. subtilis*. Biofilm formation was noted to decrease by 90% against *S. aureus* and by 97% against *E. coli* [56].

**Figure 2:**
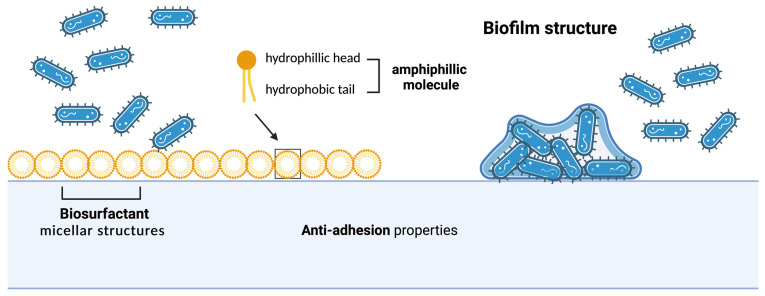
Structure of biosurfactant micelles and mechanism of action in inhibition of biofilm formation on surfaces.

Previous studies have shown that the interaction of biosurfactants with different surfaces can affect their hydrophobic properties, affecting microorganisms’ adhesion abilities and consequent biofilm formation (Figure 2) [21]. Sophorolipids show bactericidal properties when compared to conventional antimicrobial agents with bacteriostatic effects [45]. The activity shown by these compounds against biofilms could be associated with the hydrophobicity of the molecule, as well as the presence of a positive charge. An innovative approach to prevent the appearance of biofilms could be the pre-coating of medical devices, as was observed by coating medical-grade silicone discs with purified sophorolipid; an inhibitory effect against *Staphylococcus* spp. biofilm was achieved after two hours of exposure, reducing microbial cell attachment by 75% [57]. Rhamnolipids also play an important role in the disruption of biofilms. Similar to that observed in sophorolipids, the suitability of rhamnolipids to prevent microbial colonization of medical-grade instruments has also been investigated [53]. An investigation of the physicochemical and biological properties of rhamnolipids carried out by Ramos Da Silva (2019) found that the antibiofilm activity of these compounds is concentration-dependent. It was demonstrated that the treatment of *C. albicans* and *C. parapsilosis* biofilms with the mono-RL compounds at a concentration equal to twice the MIC (15.6 μg/mL) resulted in an approximate 50% reduction in cell viability (MIC50). When the concentration was increased to a value of 10 times the MIC, a reduction of 75% was observed [58].

## 4. The Use of Biosurfactants in COVID-19

The widespread spread of COVID-19 has altogether changed social and sanitation practices worldwide [59]. In this instance, the use of biosurfactants in potentially managing future pandemics validates extensive research due to their potential applications in more effective sanitation practices, drug delivery systems, and anti-viral effects [60].

To date, only emergency treatment is available for COVID-19 for patients who are at the highest risk of becoming critically ill, and therefore significant emphasis has been placed on the requirement of minimizing the spread of infection [61]. Measures such as social distancing, isolation, and personal hygiene, particularly the effective sanitation of hands, have become standard practice in the day-to-day life of most people [62]. The hands are the primary body parts that interact with the environment and are therefore susceptible to spreading infection. To eliminate pathogens, harsh agents such as synthetic surfactants and alcohol-based sanitizers are commonly utilized [41]. Recent research carried out by the Centers for Disease Control (CDC) has revealed that traditional soap is more effective than hand sanitizers or water alone when washing hands (Centers for Disease Control and Prevention, 2020).

Following the CDC recommendation, the use of alcohol-based hand sanitizers containing at least 60% alcohol is still indicated when soap is unavailable; however, the extended and frequent use of alcohol-based products can cause a negative effect on human skin resulting in discoloration and irritation [63]. Biosurfactants have been presented as a sustainable, non-toxic alternative to traditional disinfectants [59]. The amphiphilic micellar nature of biosurfactants makes them promising candidates for use in household cleaners, soaps, cosmetic products, and moisturizers [2]. Only in the past ten years, numerous biotechnological companies have focused on the development of new formulations containing biosurfactants (Evonik, 2022), and, since the outbreak of the pandemic, the optimization of cost-effective production processes has allowed biosurfactants to become commercially available in household cleaning products, detergents, and disinfectants [59]. Since 2019, companies such as Evonik (Essen, Germany), Ecover (Malle, Belgium), and Henkel (Düsseldorf, Germany) have introduced sophorolipids in a range of their household products, soaps, and other personal care products [64]. Similarly, Unilever (London, UK), BASF (Ludwigshafen, Germany), and Evonik include rhamnolipids in the formulation of detergents and other cleaning agents [64,65].

**Figure 3:**
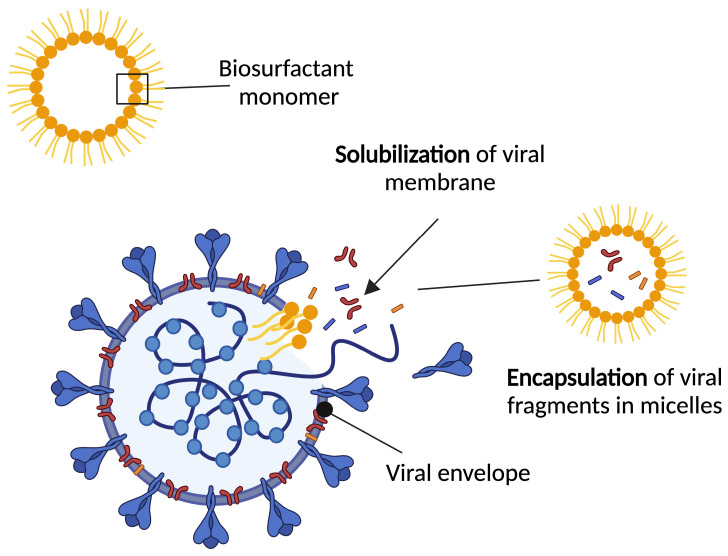
Structure of biosurfactant molecule and expected interaction with viral envelope. Mode of action relies on solubilization of the viral membrane in addition to the encapsulation of viral fragments.

The effectiveness of liquid soaps lies in the fact that biosurfactants are amphiphilic in nature: two areas of different polarity [41]. This characteristic makes them act as emulsifiers capable of dispersing one liquid into another. During hand washing, the lipophilic zone of the surfactant binds to polar molecules, such as water [60]. In the process of coronavirus inactivation, the lipophilic site of the active components of the soap is introduced into the lipid membrane of the pathogen, affecting the structural integrity and forming micellar structures (Figure 3) [59]. These self-aggregating structures can operate as effective emulsifiers and thus possess antimicrobial properties that are useful in the design of drug delivery systems [66]. Biosurfactant-based microemulsion drug delivery systems can be utilized to improve the efficacy of existing therapies by enhancing loading capacity and bioavailability or directly targeting the virus [64]. As an example of this, the inactivation of three types of viruses (HSV-1 and HSV-2, SARS-CoV-2, and PV-1) was studied using various concentrations of rhamnolipids. As reported by Giugliano et al. (2021) both HSV and SARS-CoV-2 were completely inactivated at rhamnolipid concentrations of 6 µg/mL and 25 µg/mL, respectively. No activity against PV-1 was recorded, which demonstrated that rhamnolipids target enveloped virus [67]. It was later confirmed that the inactivation of the virus is achieved due to the rhamnolipid molecules blocking the viral binding site, preventing the entry of the virus and, as a result, inhibiting replication [67].

Similarly, the anti-viral properties of sophorolipids were first reported by Shah et al. (2005) and later by Azim et al. (2006) against HIV, Epstein-Barr, and influenza virus [68,69]. Shah et al. (2005) demonstrated that inactivation of HIV was achieved using sophorolipid derivatives at a concentration of 3 mg/mL [69]. More specifically, acidic sophorolipids were found to be more virucidal against HIV and Epstein-Barr virus at lower concentrations, 200 µg/mL [68,69]. HIV, Epstein-Barr, and influenza virus are all enveloped viruses, suggesting that sophorolipids may be effective anti-viral agents against the enveloped SARS-CoV-2. Although the precise anti-viral mechanism of sophorolipids is unknown, it is believed that eradication lies in the solubilization of viral membranes [55]. Micelle development around the virus and its constituents may also play a significant role in the anti-viral activities of sophorolipids [70].

## 5. Anti-Cancer Potential of Biosurfactants

Advances in strategies to treat the wide variety of cancer diseases require efficient delivery of the active compound to the nucleus of tumor cells. To date, numerous natural products and synthetic compounds have been developed as anti-cancer drugs including camptothecin, paclitaxel, doxorubicin, and platinum compounds [71]. However, anti-cancer drugs cause serious side effects in tissues and toxicity to healthy cells, making the risk/benefit ratio for the patient sometimes unfavorable. The development of new cancer therapies must take into account a requirement for reduced toxicity to healthy cells and increased selectivity against cancer cells [58].

Biosurfactants have been widely investigated for their ability to affect tumor progression and therefore act as anti-cancer agents [72]. Numerous studies have demonstrated the anti-cancer effect of biosurfactants in vitro. For example, sophorolipids have been shown to be cytotoxic in human pancreatic (HPAC), liver (H7402), lung (A549), brain (LN229, HNCG-2), esophageal (KYSE109, KYSE450), breast, cervical (HeLa), leukemic (HL60, K562), and melanoma (SK-MEL-28) cell lines in vitro [73,74,75,76,77,78]. Rhamnolipid biosurfactants have also been shown to have anti-cancer properties in cell lines including breast cancer (MCF-7), colon cancer (CaCo-2), liver cancer (HepG2), and human promyelocytic leukemia [79,80,81,82].

The separation and purification of biosurfactants into congeners are required to fully understand their individual anti-cancer effects [16]. Previous studies of the cytotoxicity of biosurfactants on cancerous and healthy cells have presented sometimes contradictory results, which is most likely to be attributed to the use of uncharacterized biosurfactant mixtures [78]. Factors such as the nature of the cells studied, the purity of the biosurfactant used, the type of congeners investigated, or the level of reduction in surface tension arising from a given concentration of biosurfactant should be taken into consideration when determining the cytotoxic effects of biosurfactants [83]. Recently, Adu and coworkers demonstrated that when treating SK-MEL-28 cell lines, highly purified glycolipids have differential effects depending on their chemical structure. It was shown that certain congeners (lactonic-SL and mono-RL) induced cell death and prevented the migration of melanomas with little effect on healthy skin cells [84].

**Figure 4:**
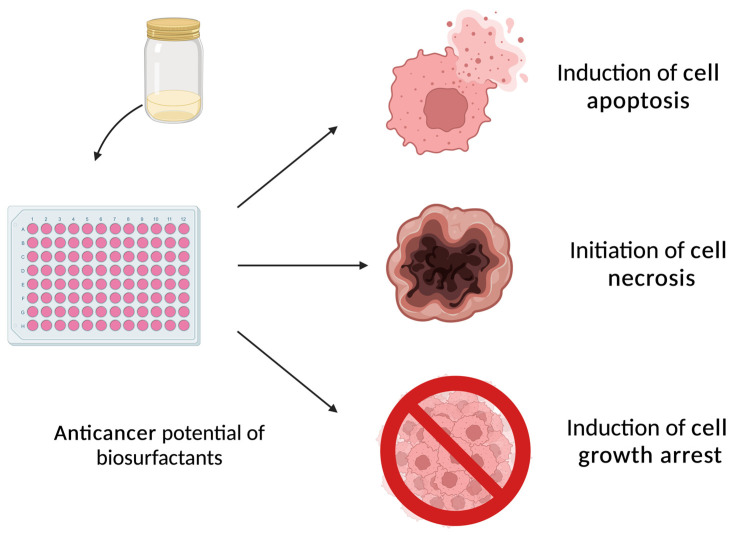
Schematic representation of the effects of biosurfactants on cancerous cells.

Even though the anti-tumor potential of these molecules is being investigated, results are still scarce, and data on the mechanisms underlying such activity are limited. As an example, glycolipids have been associated with growth arrest and apoptosis, thus inhibiting the proliferation of malignant cells (Figure 4) [85]. Callaghan et al. (2022) have shown these effects using purified acidic-SL in human colorectal cancer cell lines in Apcmin+/− mouse models. They concluded that these purified congeners induced apoptosis and necrosis, reduced migration, and inhibited colony formation in both cancer cell lines tested [86]. Another interesting example was reported by Rahimi et al. (2019), who demonstrated that rhamnolipids produced by *P. aeruginosa* MR01, when separated into congeners and purified, exhibited significant anti-cancer potential against breast cancer (MCF-7) cell lines [81]. Morphological changes and a reduction in cell viability were observed after treating the breast cancer cells for 48 h with both mono-RL and di-RL. in addition, the expression of the tumor suppressor gene p53 was increased after the treatment of both congeners, indicating the arrest of the malignant cell cycle [81].

## 6. Conclusions

Ultimately, biosurfactants have demonstrated their potential as a valuable instrument in the realm of medicine. Due to their capacity to decrease surface tension and emulsify lipophilic substances, they are valuable in biomedical applications, including drug production, drug delivery, and mitigating the toxicity of chemicals employed in medical therapies. Biosurfactants possess significant promise in infection control by aiding in the eradication or suppression of bacterial biofilms, which are prevalent in antibiotic-resistant diseases. Nonetheless, the therapeutic use of biosurfactants comes with limitations. One primary concern is the lack of standardized production methods and scalability, which restricts their cost-effectiveness and large-scale adoption. Moreover, the limited understanding of the complex biological processes and mechanisms involved in biosurfactant production restricts the optimization of these compounds for specific pharmaceutical applications. Further research is necessary, however, to comprehensively grasp the extent and potential applications of biosurfactants in medicine. Nevertheless, it is evident that biosurfactants present a promising opportunity to enhance medical treatments, mitigate drug toxicity, and combat infections with greater efficacy in the future.

## Figures and Tables

**Table 1:** Centers for Disease Control and Prevention (CDC) 2019–2022 AR Threats Report in the US [4]. Data include threat of antimicrobial resistance in the US, estimated cases, and causative organisms, including ESKAPE pathogens.

Threat	Causative Organism	Resistance	Estimate (Cases)
Urgent	***Acinetobacter***	**Carbapenem**	**7500**
	*Candida auris*	Multidrug	754
	*Clostridioides difficile*	Multidrug	202,600 *
	**Enterobacteriaceae**	**Carbapenem**	**12,700**
	*Neisseria gonorrhoeae*	Multidrug	942,000 *
Serious	*Campylobacter*	Multidrug	725,210 *
	*Candida*	Multidrug	28,100
	**Enterobacteriaceae**	**Beta-lactamase**	**197,500**
	***Pseudomonas aeruginosa***	**Multidrug**	**28,800**
	***Enterococci***	**Vancomycin**	**50,300**
	Nontyphoidal *Salmonella*	Multidrug	254,810 *
	*Salmonella* serotype Typhi	Multidrug	6130 *
	*Shigella*	Multidrug	242,020 *
	***Staphylococcus aureus***	**Methicillin**	**279,300**
	*Streptococcus pneumoniae*	Multidrug	12,000 *
	Tuberculosis	Multidrug	661
Concerning	Group A Streptococcus	Erythromycin	6200 *
	Group B Streptococcus	Clindamycin	15,300 *

* Data were extracted from 2019 AR Threats Report as some 2022 data are delayed or unavailable due to COVID-19 pandemic. Organisms in **bold** correspond to ESKAPE pathogens.

## Data Availability

Data sharing is not applicable.
